# An Efficient Approach for Lipase-Catalyzed Synthesis of Retinyl Laurate Nutraceutical by Combining Ultrasound Assistance and Artificial Neural Network Optimization

**DOI:** 10.3390/molecules22111972

**Published:** 2017-11-15

**Authors:** Shang-Ming Huang, Hsin-Ju Li, Yung-Chuan Liu, Chia-Hung Kuo, Chwen-Jen Shieh

**Affiliations:** 1 Biotechnology Center, National Chung Hsing University, 250 Kuokuang Road, Taichung 40227, Taiwan; zxzxmj2323@hotmail.com; 2 Department of Chemical Engineering, National Chung Hsing University, 250 Kuo-Kuang Road, Taichung 40227, Taiwan; judyiaa@hotmail.com (H.-J.L.); ycliu@dragon.nchu.edu.tw (Y.-C.L.); 3 Department of Seafood Science, National Kaohsiung Marine University, 142 Haijhuan Road, Nanzih District, Kaohsiung 81143, Taiwan

**Keywords:** retinol, lipase, retinyl laurate, sonication, artificial neural network (ANN), central composite design

## Abstract

Although retinol is an important nutrient, retinol is highly sensitive to oxidation. At present, some ester forms of retinol are generally used in nutritional supplements because of its stability and bioavailability. However, such esters are commonly synthesized by chemical procedures which are harmful to the environment. Thus, this study utilized a green method using lipase as a catalyst with sonication assistance to produce a retinol derivative named retinyl laurate. Moreover, the process was optimized by an artificial neural network (ANN). First, a three-level-four-factor central composite design (CCD) was employed to design 27 experiments, which the highest relative conversion was 82.64%. Further, the optimal architecture of the CCD-employing ANN was developed, including the learning Levenberg-Marquardt algorithm, the transfer function (hyperbolic tangent), iterations (10,000), and the nodes of the hidden layer (6). The best performance of the ANN was evaluated by the root mean squared error (RMSE) and the coefficient of determination (*R*^2^) from predicting and observed data, which displayed a good data-fitting property. Finally, the process performed with optimal parameters actually obtained a relative conversion of 88.31% without long-term reactions, and the lipase showed great reusability for biosynthesis. Thus, this study utilizes green technology to efficiently produce retinyl laurate, and the bioprocess is well established by ANN-mediated modeling and optimization.

## 1. Introduction

Retinol is an animal form of vitamin A that has many functions, such as the maintenance of vision, UV filters, improvement of skin aging, promotion of bone growth, maintenance of reproductive function, and can also strengthen the immune system to prevent infections [1]. Retinol and retinyl esters are major compounds of vitamin A in the body, and retinol is unstable and prone to oxidation, while the retinyl ester is not. When the retinol molecule is esterified with a long-chain fatty acid, retinyl ester is formed. Major fatty acids within the body, such as palmitic acid, oleic acid, stearic acid, and linoleic acid are involved in the esterification reaction for the synthesis of retinyl ester [2]. Moreover, the retinyl esters with long chain fatty acid only form in the body of animals [2]. Comparing with retinol obtained from foodstuffs, long-chain retinyl esters display highly lipophilic, thermal stability, and multi-processable characteristics. Considering the importance of vitamin A in human nutrition, it is also of interest to develop a convenient and economic technique to synthesize a stable form of retinol derivatives, such as retinyl esters.

Retinyl esters can be synthesized by chemical methods or enzymatic catalysis. In 2004, the bioactive retinyl ascorbates were synthesized by Abdulmajed and Heard using a chemical technique, and this method requires several steps [3]. First, the retinyl chloride was synthesized by chemical reaction of phosphorous trichloride with all-trans-retinoic acid in the dry benzene. Thereafter, ascorbic acid was added to react with retinyl chloride in the presence of potassium carbonate, and the mixture was subjected to thin-layer chromatography and column chromatography to obtain retinyl ascorbates (purity of >98.5%). Moreover, retinyl retinoate and many retinyl polyhydroxybenzoates have also been reported that they could be chemically synthesized [4,5]. A study showed that retinyl retinoate could be synthesized by performing the chemical reaction using retinol with retinoic acid in the solvent system containing a condensing agent, such as *N*,*N*-carbonyl diimidazole (CDI), and a catalyst, such as *N*,*N*-dimethylaminopyridine (DMAP) [4]. Retinyl polyhydroxybenzoates can be chemically synthesized by the base-catalyzed reaction of reactants [5]. As mentioned above, chemical synthesis of retinyl esters is a complex procedure, which is usually accompanied by increased production of byproducts, and the purification of retinyl esters requires several steps, which are often time-consuming. Most notably, these procedures are harmful to the environment because of using a large amount of organic solvent and alkaline as catalysts.

In contrast to chemical-based methods, the biosynthesis of producing natural products has been carried out in vitro by using purified enzymes, many of which have also been synthesized by organic chemists and been applied in various research fields [6]. The biocatalysis seems to be an attractive alternative to produce retinyl esters. Maugard et al. have used immobilized Novozym^®^435 to catalyze retinol and l-methyl lactate to form retinyl l-lactate, and a high yield of 90% was obtained within 24 h [7]. Although this research group also developed an enzymatic method using Novozym^®^435 to synthesize several water-soluble retinol derivatives, including retinyl adipate, retinyl palmitate, and retinyl succinate [8], there still remains some retinol derivatives, such as retinyl laurate, not synthesized by the biocatalytic methods. In such methods, enzymatic reaction products can be easily purified by filtration of the biocatalyst and evaporation of the solvent under the condition of reduced pressure, which is simpler and more environmentally friendly, as well as more economical, than that using chemical methods as mentioned above.

Many reaction conditions, including pH, kind of solvents, the quantity of water, and the method of enzyme preparation, have been suggested as the important factors on the efficiency of lipase-catalyzed reactions [9]. Considering enzyme preparation, Yin et al. have compared the catalyzing efficiency of free and immobilized lipases on retinyl palmitate synthesis, and the results showed that the immobilized form was better than the free form of the activity of catalysis [10]. Additionally, immobilized lipases have many advantages in the process of catalyzed reaction such as reusability and stability [11], so they may be the best candidate to catalyze the synthesis of retinyl esters in the industry [7,8,12].

In recent years, ultrasound technique has gained increased attention because it is advantageous for the acceleration of enzymatic reactions [13,14,15,16,17]. To the best of our knowledge, mechanical effects of ultrasound can lead to a disruption of the phase boundary between compounds, an improvement of mass transfer and a decrease in the activation energy of reactions, which could result in an accelerated rate of enzyme-catalyzed reaction [14,16]. Therefore, ultrasound-assisted immobilized lipase catalysis might be an excellent strategy for industrial biosynthesis. It has been reported that esterification efficiency of immobilized lipase on the biosynthesis of phenethyl ester [13], 4′-acetoxyresveratrol [14], ethyl butyrate [15] and d-isoascorbyl palmitate [18], can be enhanced by ultrasound assistance. However, the effect of ultrasound on the lipase-catalyzed synthesis of retinyl esters is still unknown.

Design of experiments is a set of techniques that involve investigating the effect of different variables on targeted outcomes in a controlled experiment. Response surface methodology (RSM) is one of the well-established mathematical and statistical methods for designing and building experimental models, evaluating the relative significance of each independent variable, as well as determining the optimum operating condition for the predicted responses. Within the types of RSM design, central composite design (CCD) consists of a factorial design with center points (the corners at +1 of the cube) and increases “star” points to estimate curvature, which allows the evaluation of a second-order polynomial equation [19]. Like CCD, Box-Behnken design (BBD) requires only three levels to run an experiment. It is a special three-level design of each factor that does not contain any points at the vertices of the experiment region. Now, RSM is a useful tool for understanding the interaction among various parameters, which has been applied successfully for optimizing parameters in various processes [20]. Recently, an artificial neural network (ANN) has been developed for modeling the technological process as an alternative to RSM system. ANN can easily establish the nonlinear relationship between independent and dependent variables without requiring prior knowledge of the correlation between targeted responses [21], which has made ANNs a powerful tool having higher accuracy and efficiency in the flexible fitting of experimental data, prediction, and modeling of biochemical processes, when compared to other data collection methods, such as RSM [22,23].

Taken together, retinol is a pivotal nutrient that is an important participant in various biochemical reactions; however, retinol is unstable and highly sensitive to oxidization by oxygen molecules. At present, some ester forms of retinol are generally used in nutritional supplements because of its bioavailability and stability. However, such retinyl esters are commonly synthesized by chemical procedures using a large amount of organic solvents and alkaline catalysts, easily leading to the formation of byproducts which are harmful to the environment. In this study, the aim was to develop an efficient green process with ultrasound assistance for the synthesis of retinyl laurate (stable long-chain retinyl esters), using the immobilized lipase as a biocatalyst to reduce the need for chemical solvents. In addition, we have focused especially on the optimization of reaction conditions, including reaction time, reaction temperature, enzyme concentration, and the molar ratio of reactants, modeled by CCD and an ANN, a well-known modeling/optimizing methodology for non-linear multivariate processes.

## 2. Results and Discussion

### 2.1. Effect of Ultrasound

**Figure 1:**
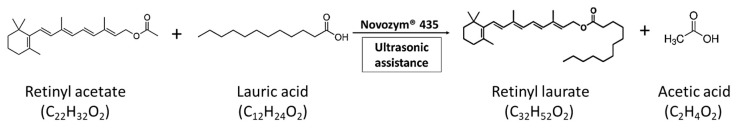
The reaction diagram of ultrasound-assisted retinyl laurate synthesis catalyzed by Novozym^®^435.

Ultrasonic assistance is increasingly becoming a quite useful method in chemical engineering-related jobs, which utilizes the energy, enhancing heat and mass transfer, for the various purposes of sample preparation. For example, Kumar et al. [24] and Yu et al. [25] investigated the performance of ultrasound on lipase-catalyzed production of fatty acid methyl esters (FAME) from soybean, which the optimum condition was finally established to obtain a high FAME yield without long-term reaction time, suggesting that ultrasonic assistance is a faster and efficient method for the production of biodiesel. Additionally, Vishwanath et al. indicated that the use of ultrasonic irradiations was in both enhancing the rate of reaction, as well as in shifting the equilibrium and resulting in a higher product yield of isopropyl esters from palm fatty acid [26]. Moreover, in our experience, the ultrasound could increase the efficiency of processes such as extraction of naturally-occurring materials, ester biosynthesis, and nutraceutical encapsulation. Thus, in this study, the catalyzing efficiency of lipase on retinyl laurate synthesis was initially examined under ultrasonic or traditional shaking (without ultrasound) conditions (Figure 1).

### 2.2. Preliminary Test

**Figure 2:**
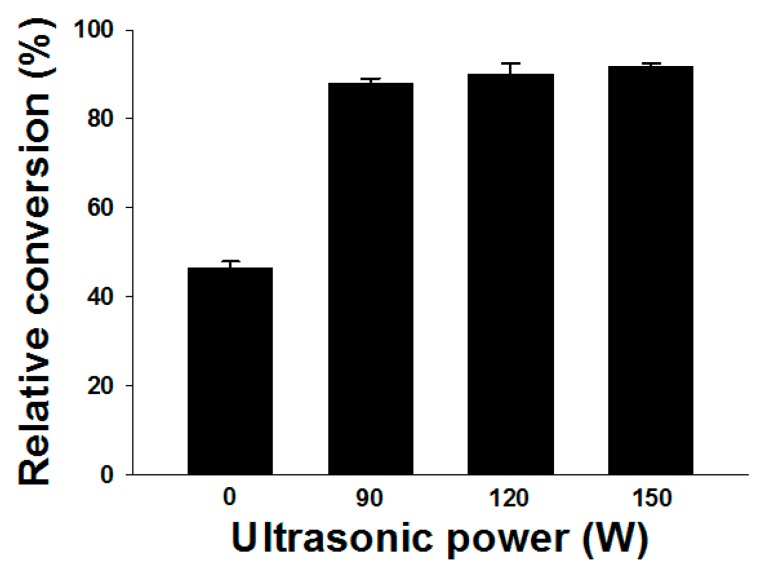
Effects of ultrasonic treatment on the lipase-catalyzed synthesis of retinyl laurate. The lipase-catalyzed synthesis of retinyl laurate was conducted under traditional shaking of 100 rpm (ultrasonic power of 0 W) or indicated intensities of ultrasonic treatment (90–150 W).

Initially, quantification of the ultrasound in enhancing the synthesis process was carried out respectively by experiments in the presence and absence of ultrasonic assistance. Our results showed that a higher relative conversion of retinyl laurate was obtained in the ultrasonic treatment group than that in traditional shaking group without sonication (ultrasonic power of 0 W) (Figure 2), suggesting that ultrasound assistance could significantly enhance the efficiency of the lipase-catalyzed reaction. In addition, an increase in relative conversion of retinyl laurate was observed when the ultrasonic power was increased in our experiment. In accordance with our findings, previous reports have suggested that ultrasound could improve immobilized lipase-catalyzed ethyl butyrate synthesis [15], and also promoted an acceleration of lipase-catalyzed acetylation [14]. In this preliminary test (under the condition of 1:10 molar ratio), although there was no significant difference between the relative conversion of retinyl laurate and ultrasonic treatment group (90–150 W), the relative conversion of retinyl laurate (50% conversion; data not shown) by ultrasonic power of 120 W is still better than that using ultrasonic power of 90 W (35% conversion; data not shown) under the condition of molar ratio of 1:1. Similar to our study, Batistella et al. [27] indicated that a high yield (~90% conversion) of lipase-catalyzed transesterification of soybean oil could be obtained at the mild irradiation power supply (~100 W), temperature (60 °C), and short reaction time (4 h). Moreover, some studies indicated that ultrasonic power could not be necessary, as a parameter, for the optimization of ultrasound-assisted enzymatic synthesis [24,25,26,27,28]. Thus, we considered that the following experiments should only be conducted by treatment with the ultrasonic power of 120 W for further process optimization.

**Figure 3:**
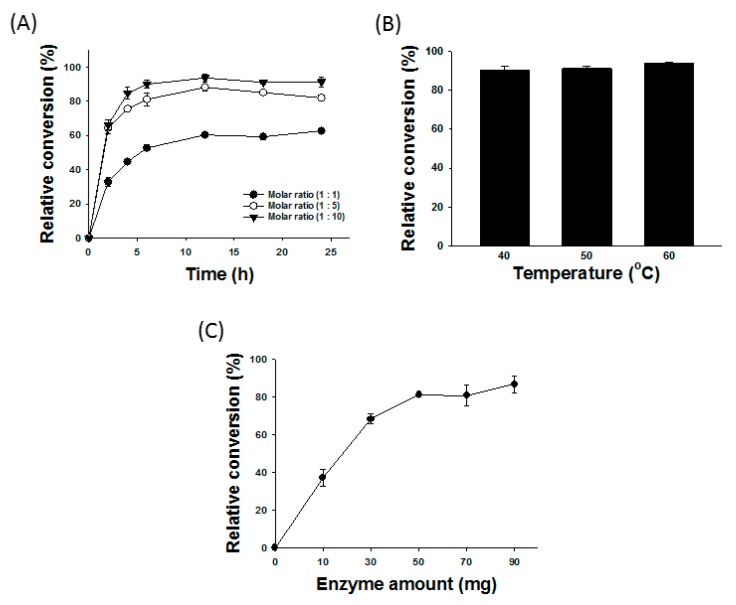
Individual effects of selected experimental parameters on relative conversion of retinyl laurate. (**A**–**C**) Effects of reaction time and the molar ratio (**A**); temperature (**B**); and enzyme amount (Novozyme^®^435) (**C**) on relative conversion of retinyl laurate. Molar ratio means the ratio of retinyl acetate (mmole) to lauric acid (mmole).

It has been reported that many reaction conditions could control the efficiency of enzymatic synthesis [9,29]. Notably, the molar ratio of substrates, reaction temperature, reaction time, and enzyme amount might have been shown to be the crucial reaction parameters in retinyl ester synthesis catalyzed by immobilized *Candida* sp. lipase [7,10,12]. Therefore, the present study further investigated the effects of these key factors on Novozym^®^435-catalyzed synthesis of retinyl laurate with sonication assistance. The effect of reaction time on Novozym^®^435-catalyzed biosynthesis of retinyl laurate under the experimental conditions of indicated molar ratios of substrates, a reaction temperature of 40 °C, enzyme amount of 50 mg and ultrasonic power of 120 W, which was shown in Figure 3A. The relative conversion of retinyl laurate was increased to 83% at 5 h when the molar ratio of retinyl acetate to lauric acid was set as 1:10. In such condition, the relative conversion of retinyl laurate was nearly constant when the reaction time was over 6 h. The effect of reaction temperature on Novozym^®^435-catalyzed biosynthesis of retinyl laurate under the experimental conditions of substrate molar ratio of (1:10), the reaction time of 3 h, enzyme amount of 50 mg and ultrasonic power of 120 W, which was shown in Figure 3B. According to the data, the relative conversion of retinyl laurate did not significantly vary at different reaction temperatures from 40 °C to 60 °C. Moreover, one study indicated that reaction temperature above 50 °C could also cause inactivation of the lipase [30], which might attenuate reaction rate. Therefore, a constant relative conversion of retinyl laurate was observed in this experiment. Figure 3C indicates the effect of catalytic amount of Novozym^®^435 on biosynthesis of retinyl laurate under the experimental conditions of substrate molar ratio (1:10), reaction time of 3 h, reaction temperature of 40 °C and ultrasonic power of 120 W. The results showed that the relative conversion of retinyl laurate might be positively related to the amount of enzyme and the substrate molar ratio within reaction conditions, and the relative conversion reached the highest levels (about 80%) when the enzyme amount was used over 50 mg. As compared to other research [10], our present study indicated that Novozym^®^435-catalyzed biosynthesis of retinyl laurate by sonication assistance could obtain a higher conversion without long-term reactions. Moreover, in order to model the ultrasound-assisted lipase-catalyzed retinyl laurate synthesis, reaction temperature (40–60 °C), enzyme amount (10–50 mg), reaction time (2–6 h) and molar ratio of retinyl acetate to lauric acid (1:1 to 1:10) were further employed in the three-level-four-factor CCD, and the optimum reaction conditions for retinyl laurate synthesis were finally established by ANN operation.

### 2.3. Artificial Neural Network (*ANN*)

**Figure 4:**
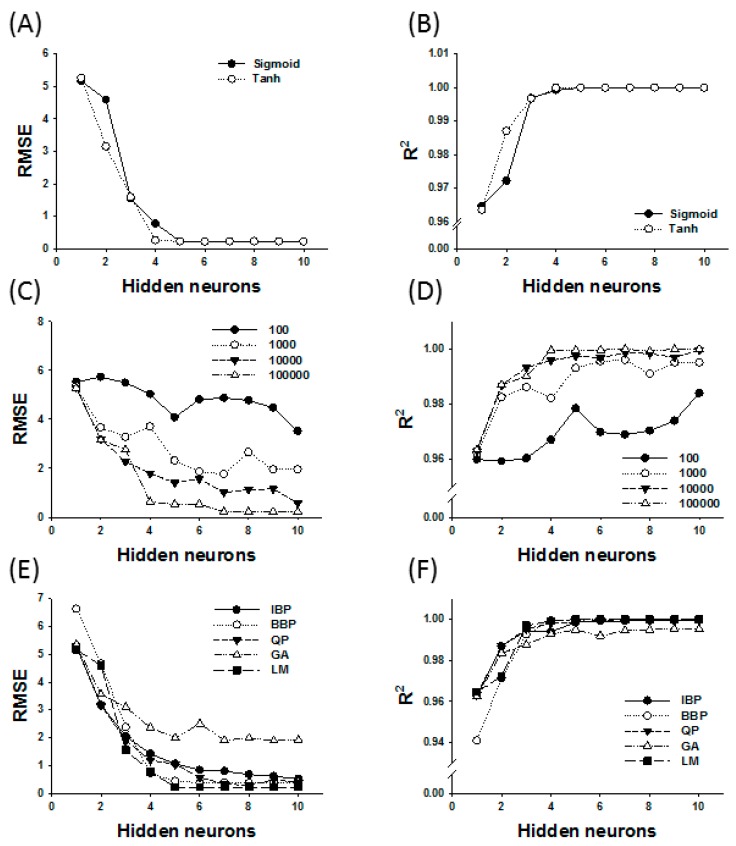
Effects of the transfer function, iteration, learning algorithm and number of hidden neurons on the performance of ANN. (**A**,**B**) Effects of sigmoid or hyperbolic tangent (Tanh) transfer functions employed in training sets on the performance of ANN; (**C**,**D**) Effect of iterations (100–100,000) on the performance of ANN; (**E**,**F**) Effects of learning algorithm and number of hidden neurons on the performance of ANN. IBP, increment back propagation; BBP, batch backpropagation; QP, quick propagation; LM, Levenberg-Marquardt algorithm; GA, genetic algorithm. ANN performance was evaluated by the coefficient of determination (*R*^2^) and the root mean square error (RMSE).

**Figure 5:**
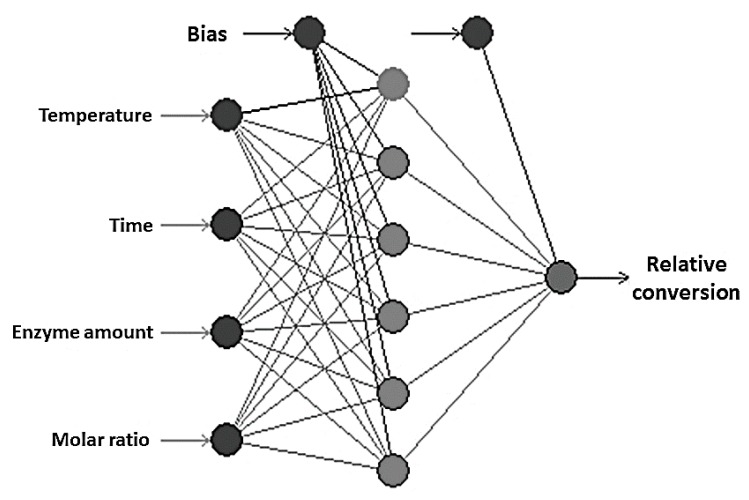
The optimal architecture of multilayer feed-forward neural network. The network contains four inputs (reaction temperature, reaction time, enzyme amount, and molar ratio of retinyl acetate to lauric acid), one hidden layer with six nodes and one output (relative conversion of retinyl laurate).

**Figure 6:**
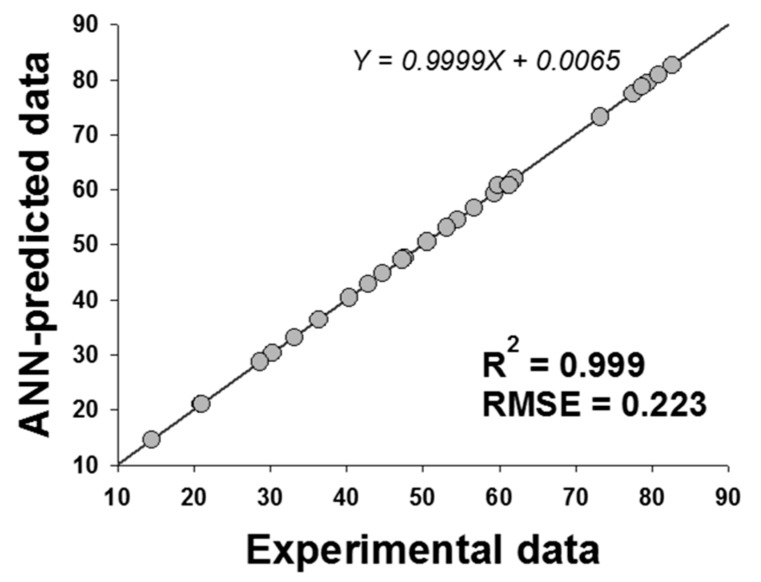
The performance of constructed ANN on data fitting. The ANN performance was evaluated by the coefficient of determination (*R*^2^) and root mean square error (RMSE), respectively. Residual values: ANN-predicted values minus experimental values.

ANN is generally known as a system with the ability to map a set of input variables into a set of outcomes without knowing the complex relationship between these parameters [31]. It has been successfully applied to investigate the possible interactions of reaction parameters and to optimize various valuable ester synthesis by lipase [32]. In this study, the data generated from the CCD (Table 1) were employed in an ANN model for the optimization of enzymatic synthesis of retinyl laurate. The various architectures for ANN models were developed using Neural Power software (CPC-X Software, version 2.5, Carnegie, PA, USA), and the best set of parameters was determined based on the maximization of *R*^2^ value and minimization of the RMSE value [33]. The proposed ANN consisted of three layers in the present work, including an input layer with four neurons (reaction time, reaction temperature, enzyme amount, and substrate molar ratio), a hidden layer with several neurons, and an output layer containing one output neuron (relative conversion). According to Figure 4, the hidden layer of ANN with six neurons can generate the best performance of the network, which exhibited lowest and stable RMSE levels and highest and stable *R*^2^ levels. Therefore, a 4-6-1 topology of the ANN was developed (Figure 5). The transfer function (Figure 4A,B), the iteration (Figure 4C,D) and the learning algorithm (Figure 4E,F) of the proposed ANN were also determined statistically based on the *R*^2^ and RMSE values. Finally, the best ANN model with a 4-6-1 topology in this study was determined to be a multilayer feed-forward connection trained by Levenberg-Marquardt (LM) algorithm using the hyperbolic tangent (Tanh) transfer function, and the iteration was set to be 10,000 to avoid overtraining and to decrease training time in this model. The learning process in this study was acquired with the values of RMSE = 0.223 and *R*^2^ = 0.999 (Figure 6).

**Figure 7:**
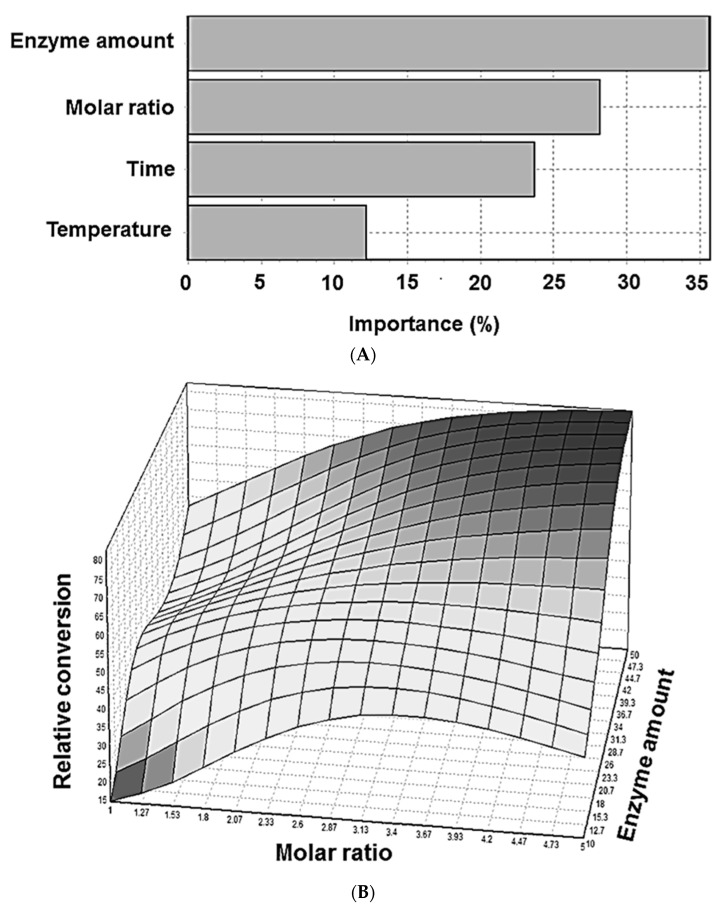
Influence of experimental variables on the lipase-catalyzed synthesis of retinyl laurate. (**A**) Importance of experimental variables in retinyl laurate synthesis. The analysis was done by Neural Power software (CPC-X Software, USA); and (**B**) the response surface plot of the ANN showing the relationships between the relative conversion of retinyl laurate and reaction parameters (molar ratio and enzyme amount).

According to Figure 7A, both enzyme amount and molar ratio exhibited great effects on the relative conversion with a relative importance around 36% and 29%, respectively. These results are consistent with the response surface plot shown in Figure 7B. In accordance with previous reports, the enzyme amount is also determined to be the most important factor in Novozym^®^435-catalyzed reaction [9,29].

In order to validate and test the predicted ability of ANN model, a completely new set of three experiments was carried out within the experimental range, which did not belong to the datasets of the design. The experimental and predicted values of the responses for ANN model are given in Table 2. The performance of newly-constructed ANN model was statistically measured based on the values of *R*^2^ and RMSE. The RMSE value for ANN was found to be 3.380, and the *R*^2^ value was found to be 0.992. From the results, it was observed that the agreement of predictive ability of ANN and experiment was found to be great. The previous report also indicated that ANN displayed a great data-predicting ability in the lipase-catalyzed synthesis of palm-based wax ester [34]. Additionally, the result showed that *R*^2^ value calculated between the actual and ANN-predicting responses were equal to 1 [34], suggesting that the ANN is really a good tool to model and optimize the process of enzymatic synthesis.

As shown in Table 3, the optimization for the lipase-catalyzed synthesis of retinyl laurate determined by Neural Power software was operated by the following conditions of the reaction time of 4.4 h, a reaction temperature of 58 °C, enzyme amount of 50 mg and substrate molar ratio of 1:5. Finally, the response performed within the optimum experimental conditions could actually obtain a relative conversion of 88.31% related to the ANN-predicted value of 84.8%, indicating that the ANN model could effectively help predict and optimize the process/parameters of ultrasound-assisted lipase biosynthesis by Novozym^®^435 to obtain a high yield of retinyl laurate.

### 2.4. Enzyme Reusability

**Figure 8:**
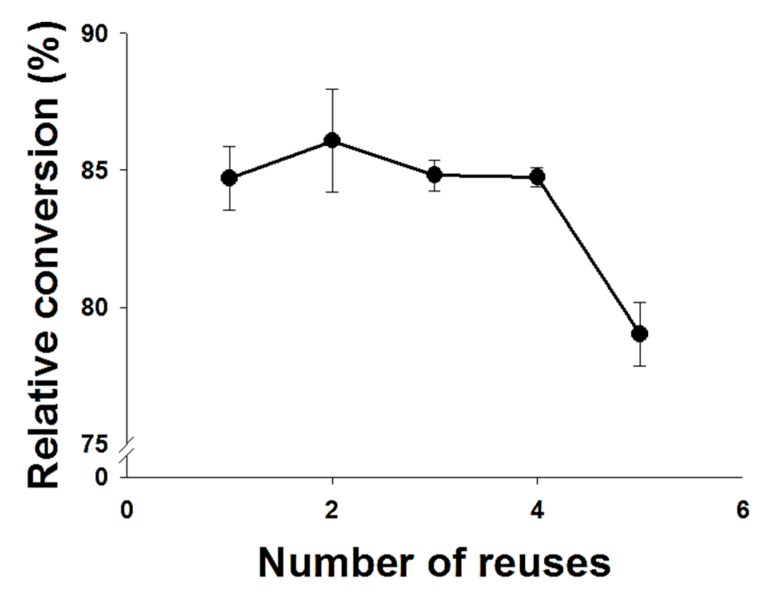
Reusability of Novozyme^®^435 on the synthesis of retinyl laurate. Reusability of Novozyme^®^435 was evaluated under optimized experimental conditions (reaction time of 4.4 h, a reaction temperature of 58 °C, an enzyme amount of 50 mg, and a molar ratio of 1:5).

The enzyme reusability has been a matter of great concern when using immobilized lipases as practical catalysts in industry. Therefore, the enzyme reusability of immobilized lipase for biosynthesis was also investigated under the optimized experimental process by determining the relative conversion of retinyl laurate (Figure 8). The immobilized lipase was recovered from the medium after the experimental reaction, followed by reusing the lipase in the next batch [33]. After five times of reuse for immobilized lipase used in this study, more than 80% of the relative conversion of retinyl laurate was still obtained. Therefore, the catalytic activity did not decrease markedly after reusing five times, indicating that an immobilized lipase, such as Novozym^®^435, could be effectively applied for retinyl laurate synthesis. According to numerous studies [35,36,37] and our experience, we determine that the lipase activity for reuse might depend on some factors, such as the solubility of the ester, reactants, usage of solvents, reaction temperature/time, and the process/method of reuse. For example, the esterification of 2-ethyl hexanol showed that after 10 cycles the enzyme retained 90% of its activity, however, the system consisting of ascorbic acid, palmitic acid, Novozym^®^435 and *tert*-butanol showed that a reduction in enzyme activity was accompanied by a reduction in reaction conversion [38]. Additionally, when we work with the immobilized lipases as catalysts for acidolysis or esterification reactions, we often wash the used enzyme as soon as possible to minimize the decrease in the enzyme activity, and the enzyme after use is washed with a suitable buffer/solvent of the reaction and the washed mother liquor is checked for residual substrate or product, requiring the number of washings to be optimized.

## 3. Materials and Methods

### 3.1. Materials

Immobilized lipase Novozym^®^435 was purchased from Novo Nordisk Bioindustrials Inc. (Copenhagen, Denmark). Retinyl acetate (≥99%) and lauric acid (99%) were purchased from Sigma-Aldrich (St. Louis, MO, USA). Other chemicals and reagents used were of analytical reagent grade.

### 3.2. Lipase-Catalyzed Retinyl Laurate Synthesis by Ultrasound Assistance

All reagents were dehydrated by molecular sieves (4 Å) for 24 h before use. Briefly, Novozym^®^435 was added into 1 mL hexane in a glass tube. Then, retinyl acetate and lauric acid were added to the mixture. The glass tube was sealed and placed in a temperature-controlled 40 kHz ultrasonic bath (Delta DC150H, New Taipei, Taiwan). Thereafter, the lipase-catalyzed retinyl laurate synthesis was carried out under various conditions of ultrasonic power (0–150 W), reaction time (2–6 h), reaction temperature (40–60 °C), enzyme amount (10–50 mg), and molar ratio of retinyl acetate to lauric acid (1:1 to 1:10).

**Figure 9:**
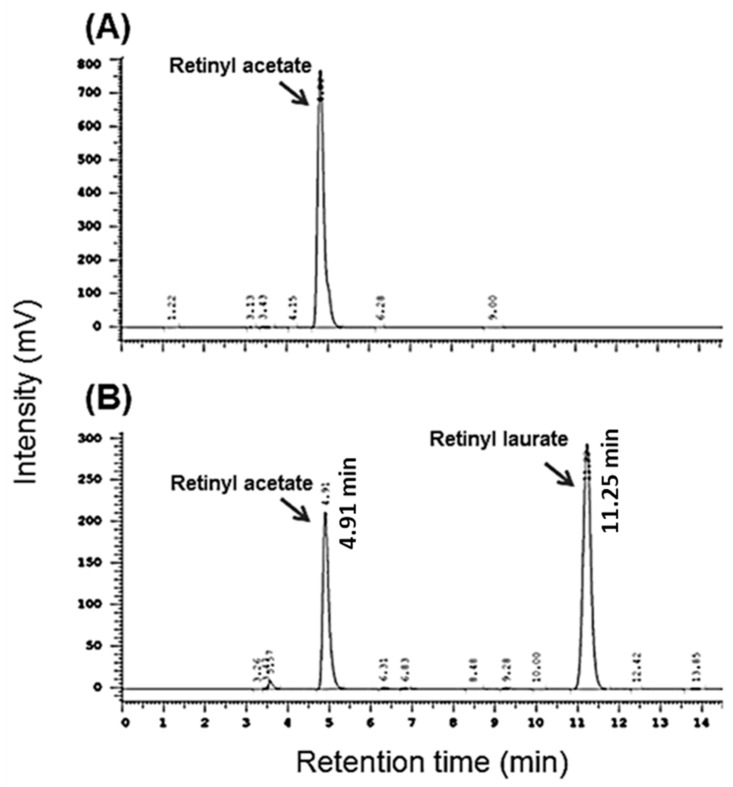
HPLC chromatogram of retinyl laurate. (**A**,**B**) show the chromatograms obtained before and after the lipase-catalyzed reaction, respectively.

At the end of the reaction, the liquid samples from reaction mixture were further withdrawn for determination of retinyl laurate by an instrument using high-performance liquid chromatography (HPLC). First, the sample was diluted and injected (20 μL) into the HPLC equipped with an ultraviolet (UV) detector (Hitachi L-7400; Tokyo, Japan) and a Thermo C18 column (250 mm × 4.6 mm, Agilent, Waltham, MA, USA). In gradient elution, all separations were carried out with a mobile phase of 0.1% acetic acid and methanol, and the flow rate was set at 1.0 mL∙min^−1^ [29]. Moreover, the retinyl laurate was absolutely detected under the condition of long-wavelength UV light at 364 nm as illustrated in Figure 9. The relative conversion was defined as (mmol of retinyl laurate production per mmol of initial retinyl acetate) × 100%.

### 3.3. Central Composite Design

A three-level-four-factor CCD for 27 experimental runs was employed in the present study. To avoid bias, the 27 runs were performed in a totally random order. The variables and their levels selected for the study of retinyl laurate biosynthesis were: reaction temperature (40–60 °C), enzyme amount (10–50 mg), reaction time (2–6 h), and the molar ratio of retinyl acetate to lauric acid, which were coded as shown in Table 4. Moreover, Table 1 shows the independent factors (*Xi*) and their levels, experimental design, and the observed data. Each experimental point was carried out in triplicate. Statistical parameters [33] performed in this study including coefficient of determination (*R*^2^) and root mean square error (RMSE) were respectively calculated by Equations (1) and (2):

(1) $$R^{2}=1-\frac{\sum_{i=1}^{n}{\left(Y_{pre}-Y_{exp}\right)}^{2}}{\sum_{i=1}^{n}{\left(Y_{m}-Y_{exp}\right)}^{2}}$$

where *Y_pre_* is the predicted yield of retinyl laurate (by ANN), *Y_exp_* is the observed yield of retinyl laurate, and *Y_m_* is the average yield of retinyl laurate; *n* is the number of experiments (*n* = 27 for CCD test and *n* = 3 for validation test).

(2) $$\mathrm{RMSE}=\sqrt{\frac{\sum_{i=1}^{n}{\left(Y_{pre}-Y_{exp}\right)}^{2}}{n}}$$

where *Y_pre_* and *Y_exp_* are the predicted and experimental yields of retinyl laurate, respectively; and *n* is the number of experiments (*n* = 27 for CCD test and *n* = 3 for validation test).

## 4. Conclusions

The present study demonstrated, for the first time, an ultrasonic system efficiently assisting with the synthesis of retinyl laurate catalyzed by immobilized lipase (Novozym^®^435). Moreover, a CCD with an ANN was operated to investigate the optimization of enzymatic synthesis. Finally, the ANN models for retinyl laurate synthesis was built, and the ANN showed good data fitting and prediction abilities. The optimal condition for ultrasound-assisted synthesis was performed by a reaction time of 4.4 h, a reaction temperature of 58 °C, an enzyme amount of 50 mg, and a substrate molar ratio of 1:5. Finally, the relative conversion of retinyl laurate of 88.31% could be actually obtained under these optimized conditions. This study successfully performed an ultrasound-assisted process by combining ANN optimization for the synthesis of retinyl laurate biocatalysis using the lipase as a catalyst to reduce the use of chemical solvents. Moreover, as compared to the biosynthetic process, our study could obtain a higher relative conversion of retinyl laurate in a shorter time. Our current evidence provides useful information for implications: (1) ultrasound-assisted biocatalysis can be an efficient strategy for producing retinyl laurate; and (2) the methodological approach using an ANN showed a great ability for predicting and optimizing the biosynthesis. Finally, it is worth emphasizing that a nutraceutical like retinol is sensitive to oxygen, which might lead to the limited practical application of chemoprevention; however, this study provides important information that the sonication-assisted biosynthesis optimized by the ANN methodology is an efficient strategy for producing targeted ester structures of nutraceuticals with enhanced stability against oxidation by air, which could be well used as supplements to promote human health.

## Figures and Tables

**Table 1:** Central composite design (CCD) experiments and observed data.

Treatment No. ^a^	Experimental Parameters				Relative Conversion (%) ^b^
	X_1_	X_2_	X_3_	X_4_	
1	2	40	30	3	33.16 ± 1.21
2	4	40	10	3	20.97 ± 1.17
3	4	40	50	3	61.99 ± 3.33
4	4	40	30	1	30.27 ± 2.67
5	4	40	30	5	56.69 ± 3.56
6	6	40	30	3	54.56 ± 1.93
7	2	60	30	3	47.59 ± 1.14
8	6	60	30	3	73.19 ± 0.74
9	4	60	10	3	50.55 ± 4.50
10	4	60	30	5	79.36 ± 0.94
11	4	60	50	3	80.85 ± 0.57
12	4	60	30	1	42.87 ± 1.41
13	2	50	10	3	21.04±1.50
14	2	50	30	5	53.10 ± 1.83
15	2	50	50	3	59.30 ± 4.06
16	2	50	30	1	28.63 ± 1.16
17	4	50	50	1	47.27 ± 0.42
18	6	50	30	5	77.47 ± 2.49
19	6	50	30	1	40.33±1.72
20	6	50	10	3	44.71 ± 1.32
21	6	50	50	3	78.69 ± 0.31
22	4	50	30	3	61.06±1.82
23	4	50	50	5	82.64 ± 1.94
24	4	50	30	3	59.80 ± 2.46
25	4	50	10	1	14.52 ± 1.85
26	4	50	30	3	61.30 ± 1.27
27	4	50	10	5	36.35 ± 3.77

^a^ The treatments were run in a random order; ^b^ Data are expressed as mean ± SD (*n* = 3).

**Table 2:** Validation of ANN-modeling retinyl laurate synthesis.

Run	Independent Variable				Relative Conversion (%)	
	X_1_	X_2_	X_3_	X_4_	Experimental ^a^	ANN-Predicted
1	4.5	46	20	4.5	60.98 ± 3.55	58.75
2	2.25	54	40	1.5	50.37 ± 2.29	46.70
3	3.25	54	15	4.5	54.91 ± 3.97	50.93
				*R*^2^		0.992
				RMSE		3.380

^a^ Data are expressed as mean ± SD (*n* = 3).

**Table 3:** An optimal trial obtained from the ANN model.

Optimal Condition				Relative Conversion (%)	
X_1_	X_2_	X_3_	X_4_	Experimental ^a^	ANN-Predicted
4.4	58	50	5	88.31 ± 0.30	84.80

^a^ Data are expressed as mean ± SD (*n* = 3).

**Table 4:** Coding of experimental parameters and related levels.

Parameters	Symbol	Coded Levels		
		−1	0	1
Reaction time (h)	X_1_	2	4	6
Reaction temperature (°C)	X_2_	40	50	60
Enzyme amount (mg)	X_3_	10	30	50
Molar ratio ^a^	X_4_	1	3	5

^a^ Molar ratio means the ratio of retinyl acetate (mmole) to lauric acid (mmole).
